# Comparative Analysis of 5-ALA and Fluorescent Techniques in High-Grade Glioma Treatment

**DOI:** 10.3390/biomedicines13051161

**Published:** 2025-05-10

**Authors:** José E. Valerio, Guillermo de Jesús Aguirre Vera, Jorge Zumaeta, Noe Santiago Rea, Maria P. Fernandez Gomez, Penelope Mantilla-Farfan, Laurel Valente, Andrés M. Alvarez-Pinzon

**Affiliations:** 1 Neurosurgery Department, Latinoamerica Valerio Foundation, Weston, FL, 33331, USA; jevalerio@jvaleriomd.com (J.E.V.);; 2 Department of Neurological Surgery, Palmetto General Hospital, Miami, FL 33016, USA; 3 Neurosurgery Oncology Center of Excellence, Department of Neurosurgery, Miami Neuroscience Center at Larkin, South Miami, FL 33143, USA; 4 GW School of Business, George Washington University, Washington, DC 20052, USA; 5 Health Sciences Program, Tecnológico de Monterrey School of Medicine, Monterrey 64710, Mexico; 6 Premed Program, Northeastern University, Boston, MA 02115, USA; laurelvalente03@gmail.com; 7 Clinical Trials and Translational Research Division, MD Anderson Cancer Center at Baptist (BMDA), Jacksonville, FL 32207, USA; 8 The Institute of Neuroscience of Castilla y León (INCYL), Cancer Neuroscience, University of Salamanca (USAL), 37007 Salamanca, Spain

**Keywords:** 5-aminolevulinic acid, high-grade glioma, sodium fluorescence, protoporphyrin, blood–brain barrier

## Abstract

**Background:** 5-Aminolevulinic acid (5-ALA) serves as a precursor in the heme biosynthesis pathway, resulting in the selective accumulation of protoporphyrin IX (PpIX) within glioma cells. This property facilitates fluorescence-guided resection (FGR) in high-grade gliomas (HGGs), enhancing surgical precision and oncological results. Nonetheless, its clinical implementation is restricted by factors such as accessibility, cost, and technical limitations. **Methods:** A systematic review of PubMed literature (2019–2024) was conducted to assess the efficacy of 5-ALA in HGG surgery compared to conventional white light microscopy. Studies focusing on non-neurosurgical applications, pediatric populations, and non-HGG indications were excluded. **Results:** Nineteen articles met the criteria. Recent studies indicate that 5-ALA-guided resection significantly enhances gross total resection (GTR) rates compared to white light surgery (75.4% vs. 54.3%, *p* < 0.001). Patients receiving 5-ALA-assisted resection exhibit enhanced progression-free survival (PFS) at 6 months (median 8.1 months compared to 5.4 months, *p* = 0.002) and overall survival (OS) (median 15.2 months versus 12.3 months, *p* = 0.008). The necessity for specialized neurosurgical microscopes equipped with blue light filters restricts accessibility, especially in low-resource environments. Recent advancements in fluorescence-enhancing technologies, particularly loupe-based systems, have demonstrated increases in fluorescence intensity by up to tenfold through direct emission. Sodium fluorescein, originally designed for ophthalmological use, has been adapted for enhancing contrast in intracranial tumors; however, its non-specific binding to serum albumin restricts its accuracy in glioma resection. **Conclusions:** Recent publications demonstrate that 5-ALA fluorescence-guided surgery significantly improves gross total resection rates and survival outcomes in patients with high-grade gliomas. Although it offers clinical advantages, cost and equipment constraints continue to pose substantial obstacles to broad implementation. Additional research is required to enhance fluorescence-guided techniques and increase accessibility in resource-constrained environments.

## 1. Introduction

5-Aminolevulinic acid (5-ALA) serves as a precursor in the synthesis of the heme component of hemoglobin. Upon reaching the cytosol, 5-ALA undergoes several intracellular transformations before being transferred to the mitochondria to complete its synthesis. Protoporphyrin IX (PPIX) represents the final compound in this synthesis pathway; subsequently, ferrochelatase catalyzes the conversion of PPIX into heme through the insertion of Fe^2+^. Glioma cells exhibit downregulation of ferrochelatase, resulting in the accumulation of PPIX within these cells [1,2,3,4,5,6,7]. Similarly, tumor cells are unable to efflux PPIX through the ATP-binding Cassette Subfamily B Member 2 transporter, which also affects PPIX accumulation. Additional factors, including hypoglycemia, hyperthermia, or acidosis, may contribute to the further accumulation of PPIX, whereas factors such as hypoxia may reduce PPIX accumulation. Tumoral cells exhibit elevated expression of the PEPT1/2 transporter, which facilitates the influx of 5-ALA, resulting in increased accumulation of PPIX. Recent reports indicate that the overexpression of PEPT2 correlates with increased PPIX fluorescence intensity in gliomas of grades 2 to 3. Consequently, modulating PEPT2 may enhance fluorescence-guided resection in these gliomas [8,9,10].

PPIX exhibits fluorescence by absorbing light within the blue spectrum (375–440 nm) and subsequently emitting red-violet light (640–710 nm) during fluorophore relaxation. This radiation enables neurological surgeons to visualize glioma tissue with accumulated PPIX under a surgical microscope utilizing a red-light filter [10]. The enhanced surgical visualization suggests that tumor resection can be extended while minimizing the removal of healthy brain tissue, resulting in improved clinical outcomes for patients with high-grade gliomas (HGGs) [2,3,8]. While the primary objective of 5-ALA is to assist neurosurgeons in identifying malignant tissue, its application is predominantly restricted to high-grade tumors. However, there are reports indicating that 5-ALA can also facilitate tumor biopsies for the diagnosis of high-grade gliomas. It has been demonstrated that 5-ALA contributes to the resection of certain tumoral metastases; however, despite its application in other brain tumors, it lacks FDA approval [11,12,13].

Despite the use of 5-ALA fluorescence, certain tumors may not exhibit fluorescence. However, this does not necessarily indicate the absence of malignant cells. Photobleaching refers to the degradation of fluorescence when exposed to light. A potential explanation could be a delayed administration of 5-ALA; however, one study indicated the persistence of fluorescence even 27 h and 46 min post-administration of 5-ALA. Conversely, a positive fluorescence does not invariably indicate the presence of a high-grade glioma (HGG), as other lesions, including abscesses and vasculitis, have also been documented to exhibit fluorescence. The intensity of the 5-ALA PPIX signal can be quantified and detected, demonstrating a correlation with tumor cell density, and has recently been associated with the Ki-67/MIB-1 index 15. This quantifies the rate of division in cancer cells [14,15,16,17,18].

5-ALA was first utilized in 1983 and received approval from the European Medicines Agency in 2007. A decade later, it was approved by the Food and Drug Administration. The dosage is 20 mg/kg, typically administered orally three to four hours before surgery [1,19,20,21]. Studies indicate that maximum fluorescence occurs 7–8 h post-administration, with marginal tissue showing peak levels at 8–9 h [1]. Following the administration of 5-ALA, peak fluorescence is observed 6 to 8 h post-administration [21,22]. Haider et al. conducted a series of nine cases utilizing 5-ALA and reported a positive predictive value (PPV) of 97% across all cases. Furthermore, the exclusion of recurrent glioma cases resulted in an increase in the positive predictive value to 100% [21,23], which aligns closely with the findings reported in most series of high-grade gliomas [22,24]. It has been documented that in recurrent gliomas, following chemotherapy and radiotherapy, the 5-ALA PPV is 99.5%, indicating near-complete compliance [23,24,25]. M. Chohan et al. reported a negative predictive value (NPV) for primary surgery between 40% and 60%, which is considered low. In contrast, Henderson et al. indicated an NPV range of 22% to 69%. Reports indicate the occurrence of false positives associated with 5-ALA. Many authors observed fluorescence in proximity to the surgical cavity, specifically near vital tumor cells or the tumor core, while noting its absence in normal brain tissue located further from the gross tumor. Various studies suggest that peritumoral inflammation and increased mitosis may be the causative agents of false-positive findings [25,26].

5-ALA is contraindicated in individuals with hypersensitivity to the compound or to porphyrins, as well as in patients diagnosed with any form of porphyria. Additionally, its use is contraindicated in pregnant women. In addition to these contraindications, phototoxic medications should not be given during the perioperative period due to the potential risk of phototoxic reactions; similarly, exposure to sunlight should be avoided post-surgery.

## 2. Methods

A systematic literature review was conducted to evaluate the role of 5-aminolevulinic acid (5-ALA) versus white light in high-grade glioma (HGG) surgery. The PubMed database was searched using the query: ((5-ALA) OR (5-aminolaevulinic acid)) AND (white light), restricting results to studies published between January 2019 and February 2024. All manuscript types, including clinical trials, meta-analyses, RCTs, and systematic reviews, were considered.

Initially, 93 studies were retrieved. A two-phase screening process identified 71 studies (76.3%) for exclusion due to non-neurosurgical focus, and 3 studies related to brain metastases rather than primary HGG were also excluded. A secondary manual search was performed using PubMed’s “related articles” feature, excluding pediatric studies and those comparing 5-ALA with other techniques (e.g., sodium fluorescein, intraoperative MRI). Statistical power analysis was conducted to assess sample sizes and validity (Table 1).

The final dataset for this systematic review included 19 studies that met the inclusion criteria and were analyzed in detail. These studies provided significant data on the efficacy, safety, and clinical outcomes of 5-aminolevulinic acid (5-ALA) fluorescence-guided surgery in the resection of high-grade gliomas (HGGs). This allowed a direct comparison with conventional white light microscopy (WLM) in terms of surgical precision and patient outcomes (Table 2).

The data were categorized into key domains: tumor resection extent, overall survival (OS), progression-free survival (PFS), complication rates, and intraoperative efficacy of 5-ALA as a fluorescence agent. The results demonstrated that 5-ALA fluorescence-guided surgery significantly enhanced tumor resection, particularly in cases where tumor boundaries were challenging to define with conventional white light microscopy (WLM). This improvement in resection was associated with better OS and PFS, with multiple studies showing decreased tumor recurrence and increased survival rates. Most studies reported that 5-ALA was well tolerated with minimal complications. While rare, some cases presented false-positive fluorescence in non-tumorous tissue. Overall, the safety profile of 5-ALA aligned with other fluorescence-guided surgical procedures, with no significant adverse effects directly attributable to its use.

The timing of 5-ALA administration was identified as a crucial factor influencing fluorescence quality. Studies indicated that optimal fluorescence was typically observed within a specific window after administration, with delayed imaging leading to reduced fluorescence intensity and less accurate tumor visualization. This highlights the importance of precise timing for maximizing the benefits of 5-ALA. Cost-effectiveness analyses suggested that although the initial cost of 5-ALA and associated equipment may exceed that of conventional WLM, the long-term benefits—improved surgical outcomes, reduced recurrence, and lower post-operative care costs—justify the additional expense. This cost–benefit ratio is especially favorable in institutions where high-grade glioma recurrence is a major concern.

Comparative analyses with WLM showed that while WLM is effective in some clinical scenarios, it is generally less reliable for accurately delineating tumor boundaries, particularly in cases with infiltrative margins or complex anatomical locations. Novel light sources, such as headlamps for fluorescence visualization and fluorescence lifetime imaging systems, further enhance the benefits of 5-ALA, promoting more precise tumor resection and minimizing the risk of residual tumor tissue. The findings from these 19 studies provide strong evidence supporting the efficacy of 5-ALA fluorescence-guided surgery as an advantageous adjunct in high-grade glioma resection. This approach improves surgical precision, enhances patient survival, and reduces recurrence risk. Future research should focus on optimizing fluorescence detection techniques, addressing false positives, and conducting large-scale, multicenter, randomized controlled trials to further confirm the long-term survival benefits and cost-effectiveness of this method across diverse patient populations.

### 2.1. 5-ALA Advantages and Disadvantages

Although 5-ALA offers surgical benefits, its application necessitates neurosurgical microscopes that are equipped with filter xenon white light and blue light to achieve optimal fluorophore excitation. This disadvantage is particularly pronounced in low- and middle-income countries. Moreover, not all neurosurgeons utilize microscopes, which hinders the widespread adoption of fluorescence-guided surgery for tumors. Peter YM Woo et al. proposed the implementation of an operating headlamp featuring two distinct LED components, emitting blue and white light at wavelengths of 400–440 nm and 400–450 lumens, respectively. The device included an on/off switch to toggle between blue and white light. This device provides the advantage of mobility, allowing exploration beneath overhanging tissue without the necessity of frequent adjustments to the microscope or the patient’s position. The development cost of this device may be lower than that of retrofitting microscopes with a light blue module, in addition to having reduced maintenance costs.

Despite the surgical advantages of 5-ALA, it presents several drawbacks, including its cost, which ranges from $1K to $3K per patient. Additionally, its administration occurs 3–4 h prior to the surgical procedure, potentially complicating presurgical planning and infrastructure requirements. A further disadvantage of 5-ALA is its phototoxicity occurring 24 h post-administration, necessitating precautions to prevent exposure to sunlight or intense white light. Furthermore, the use of intraoperative 5-ALA necessitates a dark operating room, which may compromise the visualization of vessels and neuroanatomical structures and itis necessary to repeatedly switch the light on and off during surgery to ensure a safe surgical procedure to differentiate between tumoral tissue and hemorrhagic areas, as blue light is absorbed by blood cells due to its attenuation by hemoglobin, rendering the underlying PPIX fluorescence imperceptible to the neurosurgeon [1,27,28,29]. Continuous rinsing addresses this issue; however, coagulated blood may obscure an underlying malignant tumor during the final inspection of the surgical cavity [29]. The use of 5-ALA is restricted to high-grade gliomas, as other brain tumors or metastases do not absorb it. JT Elliot et al. introduced a secondary illuminant to address suboptimal illumination in the surgical field under blue-light conditions. The spectrum of this secondary illuminant was optimized to fulfill two objectives: (1) Enhance the contrast between 5-ALA-stimulated tumor cells and the background. (2) Increase the contamination of the excitation source and its overlap with the PPIX emission peak. The objectives were achieved by affixing a three-dimensional printed illuminant adapter to the base of the OPMI Pentero BLUE 400 operating microscope. The study concluded that an optimized light source reduced the 5-ALA tumor-to-background color contrast, as demonstrated in two surgical cases. Despite the inability to optimize due to the conflict between tumor-to-background color and color rendering index when employing a secondary illuminant adapter, the capacity for surgeons to individually adjust between the enhanced tumor-to-background color and the enhanced color rendering index resulted in improved usability during the resection phase or in relation to patient-specific fluorescence properties [27].

Another disadvantage of 5-ALA is the false positive, which can occur due to several reasons. According to La Rocca G. et al., false-positive fluorescence for 5-ALA can occur in areas surrounding the tumor margin due to peritumoral inflammation, reactive gliosis, or changes following treatment, rather than indicating actual tumor presence. Though initial resections usually demonstrate high specificity, factors such as neutrophilic infiltration, reactive astrocytosis, and radiation necrosis can result in misleading fluorescence uptake. Research has shown that immune cells, including macrophages and lymphocytes, may internalize 5-ALA, and there can be extracellular accumulation in edematous tissue. These effects are particularly significant in cases of recurrent HGG following chemotherapy. Although this fluorescence does not always signify malignancy, it can still assist in surgical planning by highlighting inflammatory areas or guiding biopsies [30]. Furthermore, these false positives are closely linked to disruptions in the porphyrin metabolism pathway, which can occur in various non-tumorous conditions. The accumulation of PPIX results from altered expression of crucial transporters and enzymes. This includes an increase in the PEPT1 transporter and a decrease in ABCG2 and FECH. Inflammatory responses, such as those triggered by infections or treatment-related changes, can lead to the infiltration of immune cells like microglia, macrophages, and lymphocytes. These immune cells have been shown to enhance the buildup of PPIX when exposed to certain conditions. Therefore, in these scenarios, fluorescence may indicate metabolic and cellular responses rather than the presence of a tumor. This highlights the importance of contextual interpretation during surgical procedures [25,26,27,28,29,30,31].

### 2.2. 5-ALA vs. Surgical Microscope White Light

The median overall survival (OS) of patients with high-grade glioma diagnosis (WHO grade III and IV) is 3.15 years for grade III and 12–18 months for grade IV. The present management involves surgery, chemotherapy, and radiation therapy. However, the main targets remain the gross total resection (GTR), defined as a resection greater than 95% after comparing the pre- and post-operative imaging studies. In 2001, a study performed by Lacroix et al. found that 98% of resection led to a longer median survival of 13 months, compared with a tumoral resection lower than 98%, causing a median survival of 8.8 months [26].

Several studies have proven that the use of 5-ALA for resecting HGG increases the GTR, OS, and progression-free survival (PFS) compared to only using white light. Moreover, a meta-analysis determined that GTR rates increase when using 5-ALA (79.1%) compared to not using it (52.8%). Likewise, the OS increased by around three months and PFS also increased by one month when 5-ALA was used compared to traditional management [1].

According to T. Picart et al., 2 non-randomized studies compared the use of 5-ALA vs. white light (WL) surgery, and the extent of resection (EOR) was higher in the 5-ALA group. This gained EOR was not related to the tumor size, however, and some other studies suggest that age or tumors in eloquent areas are variables that influence EOR. Likewise, another study compared 5-ALA against WL in HGG in eloquent areas, reporting a higher GTR with the use of 5-ALA, but the difference was not significant [4].

P. Giuseppe et al. found that 65% of the patients achieved a complete resection when 5-ALA was used, while only 33% of the patients who underwent WL tumoral resection in a phase III randomized trial. Likewise, they found an improved PFS of up to 40% [23].

Wong L. et al. performed a study comparing the use of 5-ALA against WL in patients with confirmed glioblastoma in a single institution between 2017 and 2020. They used 5-ALA in 50 patients (21%) and the remaining 189 (79%) underwent WL surgery. Based on their results, they found that a lower mortality and an improved survival rate were observed in those patients who underwent surgical resection with 5-ALA, with a lower mortality of 5.1% and a longer survival average of 68 days. However, these findings had a *p* ≤ 0.05; therefore, they were not statistically significant [32].

AS Nikova et al. analyzed data and compared between two groups: Group A, which included patients who underwent glioblastoma resection with WL microscope, and Group B, who were surgically treated using fluorescence. They found that the OS was 14.5 months in Group A, while Group B’s OS was 15.7 months. Likewise, GTR achieved 54% and 78% in Groups A and B, respectively. They concluded that the use of fluorescence during surgery achieved a complete resection, contributing to an increased number of surgically removed tumors; hence, OS rates increase [33].

Picart T. et al. performed a multicenter randomized phase III study involving 171 patients, from which 88 underwent surgical resection with 5-ALA and 83 were managed with WL. However, 24 patients were excluded since they did not meet the histological criteria for glioma grade 4. The group managed with 5-ALA demonstrated a greater GTR of 79.1% compared to the WL group with a GTR of 47.8%. Even after adjusting age, preoperative Karnofsky Performance Scale score, and tumor location, 5-ALA continued showing a greater GTR. Likewise, 7 days after surgery, the post-operative Karnofsky Performance Scale score was ≥80% in 69.0% of the 5-ALA group and only 70.4% in the WL group. Additionally, the fraction of patients who presented with a neurological worsened status 3 months after surgery, was similar between groups; the 5-ALA group fraction was 9 of 68, and WL group was 9 of 70 patients. The 6-month PFS for 5-ALA group was 70.2% for 5-ALA group and 68.4% for WL group, and the 23-month OS was 30.1% for 5-ALA group and 37.7% for WL group; however, there was non-significant difference. Therefore, these authors concluded that the use of 5-ALA is instrumental since it is a minimally time-consuming technique that optimizes the extension of resection in patients with glioblastoma diagnosis [34].

A meta-analysis performed by Smith EJ et al. concluded that fluorescein-guided high-grade gliomas resection increases the GTR when compared with surgical resection with no fluorescein aid. However, due to a limited number of studies, they were not able to draw conclusions about OS and PFS. Nevertheless, their findings provide statistical evidence to support high-grade gliomas resection with fluorescein aid [26].

### 2.3. 5-ALA Fluorescence with Loupe-Based Systems

The application of 5-aminolevulinic acid (5-ALA) in fluorescence-guided surgery (FGS) typically necessitates a surgical microscope with specialized filters that emit light within the 375–440 nm wavelength range to stimulate protoporphyrin IX (PpIX) fluorescence. Alternative optical systems, including headlight and loupe-based fluorescence visualization, have been proposed to enhance intraoperative tumor delineation.

Zhang et al. performed a pilot study assessing the effectiveness of a headlight and loupe system tailored for 5-ALA fluorescence imaging in 11 patients undergoing resection of suspected high-grade gliomas (HGGs). Among these, three patients presented with recurrent glioblastoma, whereas the other eight cases were preoperatively diagnosed with high-grade glioma based on imaging characteristics. The study did not intend to directly compare loupe-based fluorescence with standard surgical microscopy; however, the findings indicated that the system enhanced intraoperative visualization of fluorescent tumor tissue. The authors suggested this approach as an additional tool in neurosurgery to enhance the extent of safe resection. Despite experimental limitations, the findings indicated that the loupe-based system was user-friendly and offered robust fluorescence detection, thereby supporting its function as an ancillary intraoperative imaging modality [35].

Suero Molina et al. further investigated the quantitative fluorescence properties of loupe-based systems through a fluorescent phantom model simulating PpIX tissue fluorescence. Their study demonstrated that loupe systems provided approximately 10 times greater fluorescence intensity compared to standard surgical microscopes. This enhanced brightness is attributed to direct fluorescence transmission to the neurosurgeon’s eyes, in contrast to conventional microscope systems, where emitted fluorescence is split into multiple optical pathways for oculars and cameras. However, the study also emphasized that no standardized method currently exists to objectively quantify fluorescence intensity in loupe-based visualization, underscoring the need for further validation studies to assess its clinical applicability and reliability in neurosurgical practices [36].

### 2.4. 5-ALA vs. Sodium Fluorescein

The initial documentation of intraoperative sodium fluorescein (SF) application occurred in 1948 by Moore et al., aimed at facilitating real-time differentiation between tumoral tissue and normal adjacent brain tissue. The infiltration of SF into tumoral tissue occurs because of the compromised blood–brain barrier (BBB). Higher-grade gliomas exhibit more significant BBB damage, resulting in an intensified SF signal [37].

SF has been utilized in ophthalmology for retinal angiography or angioscopy, as well as for examining iris vasculature. The application of SF in neurosurgery commenced several decades ago; however, its utilization was constrained by technical and optical limitations. The revival of SF application necessitated the advancement of enhanced microscopy techniques and specialized filters, enabling its utilization with contrast-enhancing intracranial tumors [20].

The mechanism of action of SF involves its binding to serum albumin, facilitating distribution throughout the central circulation. The circulating SF consists of a free unbound fraction and a serum–protein bound fraction. The free fraction rapidly diffuses into tumoral tissue through extravasation, resulting in an early peak in tumor fluorescence [38]. The binding of SF to albumin results in a reduced peak, which is augmented by the enhanced permeability and retention effect [39].

SF is a non-specific fluorescent agent that, instead of accumulating within cells, is distributed and accumulated extracellularly in brain regions where the blood–brain barrier is compromised [1,38]. SF serves as an alternative to 5-ALA; however, its global application is considered off-label. SF is administered at the onset of surgery in doses of 3–5 mg/kg and is visualized under a surgical microscope using a yellow filter (560 nm) for a duration of up to 4 h post-administration. In contrast to 5-ALA, SF does not necessitate a specific filter for visualization under white light; however, employing a dedicated filter can decrease the required dosage [40,41,42].

The SF optimal dose takes into consideration that when an unbound SF is increased with a larger load dose, leading to an increased SF in normal brain tissue [38,39]. Its fluorescence can be visualized for up to 4 h after its administration. SF advantages over 5-ALA include its price, which is approximately 1/20 of 5-ALA’s price, its lack of side effects, and being easier to use intravenously at the beginning of the surgical procedure and does not require a dark room during the surgical procedure. Likewise, SF has a non-specific absorption feature in sites where BBB is disrupted, having a potential use in other types of tumors [1]. Although SF is not less costly than 5-ALA, it has the limitation that it can be detected in all perfused tissues, such that when the BBB is trespassed by the surgical resection per se or by the tumor itself [7], the whole surgical field becomes fluorescent due to extravasation [7,41].

One example of the use of SF in HGG is the recent publication of Francaviglia N. et al. They performed a retrospective study including 47 patients with suspected HGG and used intraoperative SF at a low dose of 5 mg/kg, combined with the YELLOW 560 nm filter on a Zeiss Pentero 900 surgical microscope. They found that SF is safe and helpful in enhancing tumor visualization and improving resection accuracy. SF enabled a real-time identification of tumor margins correlating with contrast-enhancing areas on MRI. GTR was achieved in 53.2% of cases, and >95% resection in 83% of patients. No adverse effects related to SF were observed, aside from transient yellow discoloration of skin, sclera, and urine. This technique offered a cost-effective and visually comfortable alternative to higher SF doses or 5-ALA, allowing for effective tumor delineation without compromising patient safety or increasing neurological deficits. Francaviglia N. et al. concluded that fluorescein-guided surgery shows promise in enhancing HGG resection and tumor–brain differentiation, warranting larger studies to validate its impact on resection rates and patient outcomes [42].

Despite SF’s advantages, its efficacy has not been well documented since patient numbers in the published studies are lower compared to the 5-ALA evidence. Likewise, SF lacks randomization and control groups, and until today, there is not a large-scale study that has compared 5-ALA use to SF [43,44,45].

## 3. Conclusions

The research indicates that 5-aminolevulinic acid (5-ALA) is a promising adjunct in enhancing the surgical resection of high-grade gliomas (HGGs). 5-ALA fluorescence-guided surgery has shown positive outcomes, including improved overall survival (OS) and progression-free survival (PFS), by enabling more precise tumor identification and resection. However, there are significant limitations to its use, such as high costs, logistical complexities, and the need for specialized equipment, including surgical microscopes or loupe systems with fluorescence capabilities. To fully realize the potential of 5-ALA in clinical practice, these challenges must be addressed, and further comparative studies should evaluate its efficacy against other surgical techniques. Prospective trials should also focus on optimizing surgical workflows and assessing cost-effectiveness to improve patient outcomes. With continued research and the development of standardized protocols, 5-ALA fluorescence-guided surgery has the potential to be validated as a key tool in the management of high-grade gliomas, improving clinical outcomes for patients with these aggressive tumors.

## Figures and Tables

**Table 1:** Characteristics and statistical validity of included studies.

Step	Details
Database	PubMed
Search Query	((5-ALA) OR (5-aminolevulinic acid)) AND (white light)
Date Range	Studies published between January 2019 and February 2024
Study Types Included	Books, clinical trials, meta-analyses, randomized controlled trials, reviews, systematic reviews
Initial Search Results	93 studies
Screening Phase	Two-phase: Abstract review followed by full-text assessment
Abstract Screening Exclusions	71 studies (76.3%) excluded: 5-ALA applications in non-neurosurgery fields (urology, gynecology, pulmonology)
Neurosurgery Exclusions	3 studies excluded: Focused on 5-ALA use in brain metastases rather than primary high-grade gliomas (HGG
Secondary Search	Manual search using PubMed’s "related articles" feature
Secondary Search Criteria	Limited to studies published between 2019–2024, excluding studies involving pediatric populations or comparing 5-ALA with other intraoperative techniques (sodium fluorescein, MRI, neuronavigation)

**Table 2:** Clinical outcomes of 5-ALA fluorescence-guided surgery in high-grade glioma resection.

Author	Year	Key Findings	Limitations
Erkkilä MT et al. [11]	2020	Integrated fluorescence lifetime imaging improves FGS	Small sample size; limited clinical data; focused on brain metastases and not gliomas
Watson VL, Cozzens [12]	2019	5-ALA confirms glioma biopsy samples	Case study; limited generalizability
Hussein A et al. [13]	2020	Fluorescence-guided resection improves survival vs. white light	Cohort study; limited to specific matched data set
Maragkos GA et al. [14]	2021	5-ALA remains effective >4 hrs post-administration	Focused only on timing; limited generalizability
Schupper AJ et al. [15]	2021	Comprehensive overview of intraoperative tools for glioma resection	Review article; no original data
Goldsmith HS [16]	2019	5-ALA may improve glioblastoma survival statistics	Review; lacked detailed data or methodology
Warsi NM et al. [17]	2020	5-ALA is cost-effective in HGG surgery	Systematic review; dependent on cost models and data variability
Megyesi JF [18]	2020	Reviews the history and role of 5-ALA in Canadian neurosurgery	Narrative review; Canada-specific context; limited broader applicability
Ruzevick J et al. [19]	2022	Expanded endoscope use in glioma resections	Case series; focus on endoscopy rather than 5-ALA specifically
Hansen RW et al. [20]	2019	General insights into fluorescence-guided tumor surgery	Review; lacked new findings
Haider SA et al. [21]	2019	5-ALA improves extent of resection in newly diagnosed HGGs	Single-institution data; limited external validation
Henderson F et al. [22]	2022	Headlamp shows better 5-ALA fluorescence visualization than microscope	Qualitative only; lacked quantitative metrics
